# Development of an Automated Chemiluminescence Assay System for Quantitative Measurement of Multiple Anti-SARS-CoV-2 Antibodies

**DOI:** 10.3389/fmicb.2020.628281

**Published:** 2021-01-15

**Authors:** Sousuke Kubo, Norihisa Ohtake, Kei Miyakawa, Sundararaj Stanleyraj Jeremiah, Yutaro Yamaoka, Kota Murohashi, Eri Hagiwara, Takahiro Mihara, Atsushi Goto, Etsuko Yamazaki, Takashi Ogura, Takeshi Kaneko, Takeharu Yamanaka, Akihide Ryo

**Affiliations:** ^1^Department of Microbiology, Yokohama City University Graduate School of Medicine, Yokohama, Japan; ^2^Department of Pulmonology, Yokohama City University Graduate School of Medicine, Yokohama, Japan; ^3^Bioscience Division, Reagent Development Department, Tosoh Corporation, Kanagawa, Japan; ^4^Advanced Medical Research Center, Yokohama City University, Yokohama, Japan; ^5^Life Science Laboratory, Technology and Development Division, Kanto Chemical Co., Inc., Kanagawa, Japan; ^6^Department of Respiratory Medicine, Kanagawa Cardiovascular and Respiratory Center, Yokohama, Japan; ^7^Department of Health Data Science, Yokohama City University Graduate School of Data Science, Yokohama, Japan; ^8^Clinical Laboratory Department, Yokohama City University Hospital, Yokohama, Japan; ^9^Department of Biostatistics, Yokohama City University Graduate School of Medicine, Yokohama, Japan

**Keywords:** SARS-CoV-2, COVID-19, quantitative serological test, multiple antibody measurements, automated chemiluminescent enzyme immunoassay

## Abstract

**Objectives:**

Serological tests for COVID-19 have been instrumental in studying the epidemiology of the disease. However, the performance of the currently available tests is plagued by the problem of variability. We have developed a high-throughput serological test capable of simultaneously detecting total immunoglobulins (Ig) and immunoglobulin G (IgG) against nucleocapsid protein (NP) and spike protein (SP) and report its performance in detecting COVID-19 in clinical samples.

**Methods:**

We designed and prepared reagents for measuring NP-IgG, NP-Total Ig, SP-IgG, and SP-Total Ig (using N-terminally truncated NP (ΔN-NP) or receptor-binding domain (RBD) antigen) dedicated automated chemiluminescent enzyme immunoassay analyzer AIA-CL1200. After determining the basal thresholds based on 17 sera obtained from confirmed COVID-19 patients and 600 negative sera, the clinical validity of the assay was evaluated using independent 202 positive samples and 1,000 negative samples from healthy donors.

**Results:**

All of the four test parameters showed 100% specificity individually (1,000/1,000; 95%CI, 99.63–100). The sensitivity of the assay increased proportionally to the elapsed time from symptoms onset, and all the tests achieved 100% sensitivity (153/153; 95%CI, 97.63–100) after 13 days from symptoms onset. NP-Total Ig was the earliest to attain maximal sensitivity among the other antibodies tested.

**Conclusion:**

Our newly developed serological testing exhibited 100% sensitivity and specificity after 13 days from symptoms onset. Hence, it could be used as a reliable method for accurate detection of COVID-19 patients and to evaluate seroprevalence and possibly for surrogate assessment of herd immunity.

## Introduction

Severe acute respiratory syndrome coronavirus 2 (SARS-CoV-2) is the causative agent of the coronavirus disease 2019 (COVID-19). The ongoing global pandemic caused by SARS-CoV-2 is one of the most serious crises that humanity has faced in the recent past. COVID-19 infections require intensive treatment in 20–25% of patients and result in death of 4–11% of hospitalized patients (Singhal, 2020). Serological tests are particularly helpful in the detection of ongoing COVID-19 infection, depending on the time course of the illness. Moreover, serological assays enable to estimate the prevalence of existing immunity to SARS-CoV-2 in a population (Fontanet and Cauchemez, 2020).

In general, antibody testing not only helps to indirectly diagnose an infection but also reflects the individual immune response (Manners et al., 2020). This is fundamentally different from reverse transcription polymerase chain reaction (RT-PCR) or antigen detection tests, which measure viral nucleic acids and antigens, respectively. Unlike other infections, COVID-19 exhibits concurrent elevation of immunoglobulin (Ig) G and IgM in the second week after disease onset, with IgM reaching peak titers in the second week and IgG in the third week of the disease (Long et al., 2020a; Sun et al., 2020). Since the transition of antibody titers over time is different in different types of immunoglobulin, it is recognized that the measurement of multiple antibody titers can holistically assess the immune status in infected individuals.

Various antigens of SARS-CoV-2 can be utilized by serodiagnostic kits for the detection of virus-specific antibodies. These include viral surface antigens such as membrane proteins, envelope proteins, and spike protein (SP) and non-surface antigens such as the nucleocapsid protein (NP) (Masters, 2006; Benvenuto et al., 2020). Of these, the SP and the NP are the most widely employed for serological diagnosis (Ma et al., 2020). The SP mediates viral entry into host cells through the receptor-binding domain (RBD) and antibodies against RBD are predicted to represent the protective immunity to SARS-CoV-2 infection (Poh et al., 2020; Robbiani et al., 2020). The NP not only participates in ribonucleic acid (RNA) packaging and virus particle release but also plays a principal role in orchestrating various intracellular events required for viral replication after entry (McBride et al., 2014). The NP shares antigenic similarity with other human corona viruses and may exhibit cross-reactivity when used in serological tests as the full-length protein, but is highly specific for SARS-CoV-2 in the absence of its N-terminal amino acid residues (Guo et al., 2020: Yamaoka et al., 2020).

High-throughput COVID-19 serology testing is required for epidemiological surveillance. The most common method for measuring antibody titers is enzyme-linked immunosorbent assay (ELISA). Although ELISA has many advantages, it is a time-consuming and labor-intensive procedure with a higher likelihood of technical errors than other automated platforms (Wang et al., 2017). To overcome these problems, we used the automated chemiluminescent enzyme immunoassay system AIA-CL1200 (TOSOH, Japan), to develop a serodiagnostic test that quantitatively detects SARS-CoV-2 antibody titers. Apart from lower manual errors, this platform also has the inherent advantage of being more precise and sensitive than ELISA (Cinquanta et al., 2017). We evaluated the performance of the AIA-CL1200, and analyzed chronological change of antibody titers in patients.

## Materials and Methods

### Antigen Design

Previously, we showed that the cross-reactivity of NP is due to conserved residues in its N-terminus; accordingly, truncated N-terminal NP (ΔN-NP) is highly specific for detection of SARS-CoV-2. ΔN-NP (121–419 aa) was synthesized using the wheat germ cell–free protein synthesis system and purified using Ni-Sepharose beads (Yamaoka et al., 2020). The complementary deoxyribonucleic acid (cDNA) sequence corresponding to the RBD (residues 319-541) of SARS-CoV-2 spike protein was chemically synthesized and inserted into in-house Strep-tagged mammalian expression vector. Strep-tagged RBD protein was transient expressed in a mammalian cell (Expi293, Thermo Fisher Scientific), purified by Strep-tag purification system (IBA) (Schmidt and Skerra, 2007).

### Development of Antibody Detection Reagents and Calibration of AIA-CL1200

We developed four types of serological test reagents (NP-IgG, NP-Total Ig, SP-IgG, SP-Total Ig test) to detect anti–SARS-CoV-2 antibodies using the AIA-CL1200 automated chemiluminescence system. The serological test comprises of an automated immunoassay for the quantitative determination of either serum IgG or Total Ig (IgG, IgM, IgA, IgE, etc.) levels against the ΔN-NP or RBD antigen. Micro-magnetic beads coated with ΔN-NP or RBD antigen (0.10 mg/ml, 75 μl) were added to the first reaction layer, and alkaline phosphatase–conjugated anti-human IgG or ΔN-NP antigen (1.40 mg/ml, 50 μl) or RBD antigen (0.70 mg/ml, 50 μl) was added to the second reaction layer and prepared by an immediate lyophilization in a test cup. The AIA-CL1200 continuously performs the automated analysis process that includes specimen dispensing reaction cup, incubation of the reaction cup, bound/free washing procedure, dispensing of chemiluminescent substrates (DIFURAT), chemiluminescence detection, and reporting of index value, all together in 15 min (up to 240 tests/h/device with the AIA-CL2400, 120 tests/h/device with the AIA-CL1200). For NP-IgG and SP-IgG test, 10 μL of serum was used, which underwent an automated dilution (1:10) with dedicated diluent buffer (SD-Z). For NP-Total Ig and SP-Total Ig test, 20 μL of serum was used directly without dilution. We measured the standard sera for each lot of assay reagents and formulated the calibration curve.

### Patient Cohorts

For initial performance evaluation, 600 samples of healthy donors collected prior to the spread of COVID-19 infection (purchased from TRINA BIOREACTIVES AG, Zurich, Swiss) and 17 samples from COVID-19 antibody–positive patients (according to the Abbott Architect SARS-CoV-2 IgG antibody test) were purchased from Biomex GmbH (Heidelberg, German). To examine the correlation between the results of the NP-IgG and NP-Total Ig tests from this study with those of other commercially available kits, 29 samples from COVID-19 antibody–positive patients were purchased from Biomex GmbH. All 29 samples had already measured by Abbott NP-IgG kit and 11 of 29 samples had already measured by Roche NP-Total Ig. We measured all 29 samples of NP IgG and NP-Total Ig with AIA-CL1200. Since it was difficult to obtain samples that have been already found positive by quantitative kit of antibodies against SP available from other companies, we did not compare the result of SP-IgG and SP-Total Ig with other companies. For calculation of clinical sensitivity and specificity, a total of 202 samples from 44 symptomatic patients with PCR-confirmed SARS-CoV-2 infection hospitalized in Yokohama City University Hospital (YCUH) and Kanagawa Cardiovascular and Respiratory Center (KCRC) were used. The severity of COVID-19 on admission was determined according to National Institutes of Health (NIH) classification (National institutes of health, 2020). If the patient was in the recovery phase (more than 30 days after symptoms onset and the symptoms were improving on admission), categorized as “others.” To ensure the quality of the clinical specimens, one patient with a history of anticancer therapy was excluded. One thousand samples of healthy donors collected prior to the spread of COVID-19 infection (provided by the Biobank Division of Yokohama City University Advanced Medical Research Center) were used as negative controls.

Mann-Whitney Rank Sum test was performed to identify the correlations of health donors and COVID-19 patients. In the initial evaluation, we set the cutoff values of NP-IgG, NP-Total Ig, SP-IgG and SP-Total Ig titers by maximizing the overall prediction performance with the Youden’s J index (J = sensitivity + specificity - 1) based on the receiver operating characteristic (ROC) analysis, and defined them as 1.0 index. Next, the sensitivity, specificity and 95% confidence interval (95% CI) of the clinical samples was calculated using ROC curve. A *p*-value of < 0.05 was considered to be indicative of statistical significance. All statistical analyses were performed by using GraphPad Prism 6 software.

All healthy donors provided written informed consent for use of their clinical and biological data for the purpose of the scientific research. All patients were recruited using the opt-out approaches. The study was performed according to protocols approved by our institutional review board.

## Results

### Preparation of Reagents and Determination of the Optimal Cutoff Value

**FIGURE 1:**
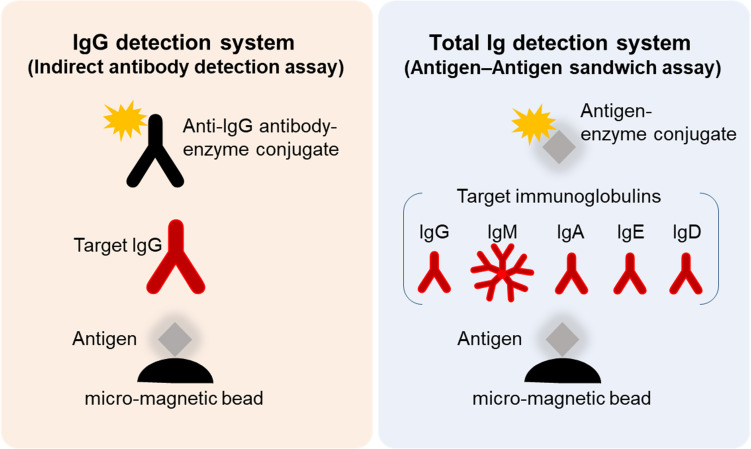
Schematic illustration of SARS-CoV-2 NP-IgG, NP-total Ig, SP-IgG, and SP-Total Ig tests dedicated AIA-CL1200. Measurement method of the NP-IgG, NP-Total Ig, SP-IgG, and SP-Total Ig tests dedicated AIA-CL1200. Micro-magnetic beads coated with ΔN-NP or RBD antigen were added to the first reaction layer. NP-IgG or SP-IgG in patient’s serum was captured by alkaline phosphatase–conjugated anti–human IgG secondary antibody (left panel). NP-Total Ig or SP-Total Ig was captured by alkaline phosphatase–conjugated ΔN-NP or RBD antigen (right panel). Alkaline phosphatase, used as the labeling enzyme, was detected using chemiluminescent substrates (DIFURAT). NP, nucleocapsid protein; SP, spike protein; AIA-CL, automated chemiluminescent enzyme immunoassay analyser; RBD, receptor-binding domain.

**FIGURE 2:**
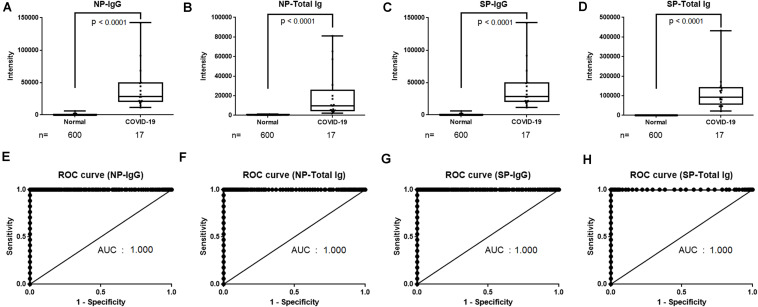
Determination of cutoff values of NP-IgG, NP-Total Ig, SP-IgG and SP-Total Ig tests dedicated AIA-CL1200. **(A)** Comparison of NP-IgG, **(B)** NP-Total Ig, **(C)** SP-IgG, and **(D)** SP-Total Ig titers between COVID-19 patients and non-infectious healthy donors in the AIA-CL1200. 17 serum samples derived from COVID-19 patients and 600 samples from non-infectious healthy donors were used for analysis. Statistical analysis was performed by Mann–Whitney Rank-Sum test, and *p*-values are presented above the box plots. **(E)** ROC curves for NP-IgG, **(F)** NP-Total Ig, **(G)** SP-IgG, and **(H)** SP-Total Ig tests as measured in **(A–D)**. Cutoff values at 1.0 index were 3,889 counts of intensity for NP-IgG and 1,705 counts for NP-Total Ig, 6,700 counts for SP-IgG, 1,000 counts for SP-Total Ig. NP, nucleocapsid protein; SP, spike protein; AIA-CL, automated chemiluminescent enzyme immunoassay analyser; AUC, Area under curve; ROC, receiver operating characteristic.

Reagents for the NP-IgG, NP-Total Ig, SP-IgG, and SP-Total Ig tests were prepared using ΔN-NP and RBD antigen, respectively. Schematic illustration of the assay principles used in the TOSOH AIA-CL system is depicted in Figure 1. We optimized the assay of AIA-CL by adjusting the amount of immobilized antigen on micro-magnetic beads, the reaction time and so on. Since antigen dosage is the most important parameter to develop antibody detection kit, in order to optimize the concentration of SP-IgG, we set up the concentrations of immobilized SP antigen on micromagnetic beads as 0.005, 0.01, and 0.30 mg/dl and measured the antibody titers of SP-Total Ig. 0.30 mg/dl of SP antigen was not appropriate because the intensity counts of two negative samples were close to those of positive samples. Finally, the dosage of immobilized SP antigen was decided to be 0.01 mg/dl (Supplementary Figure 1). After confirming within-run reproducibility, we assessed the linearity of the four antibody tests by a two-fold serial dilution of COVID-19 patient’s serum. The ratio of the intensity counts of chemiluminescence signals to serum dilution concentration (gradient value) was calculated for each sample. We found that the gradient value was almost constant representing a good linearity at lower concentration while there were saturated signals at the highest concentration in all the four tests (Supplementary Figure 2). We then measured the intensity of the chemiluminescence signals of 600 healthy donor samples and 17 COVID-19 antibody–positive samples (Figures 2A–D). Using the ROC curve, we determined the cutoff values at 3,889 counts of intensity for NP-IgG, 1,705 counts for NP-Total Ig, 6,700 counts for SP-IgG, and 1,000 counts for SP-Total Ig, and defined them as 1.0 index (Figures 2E–H).

### Chronological Analysis of Antibody Titers

**FIGURE 3:**
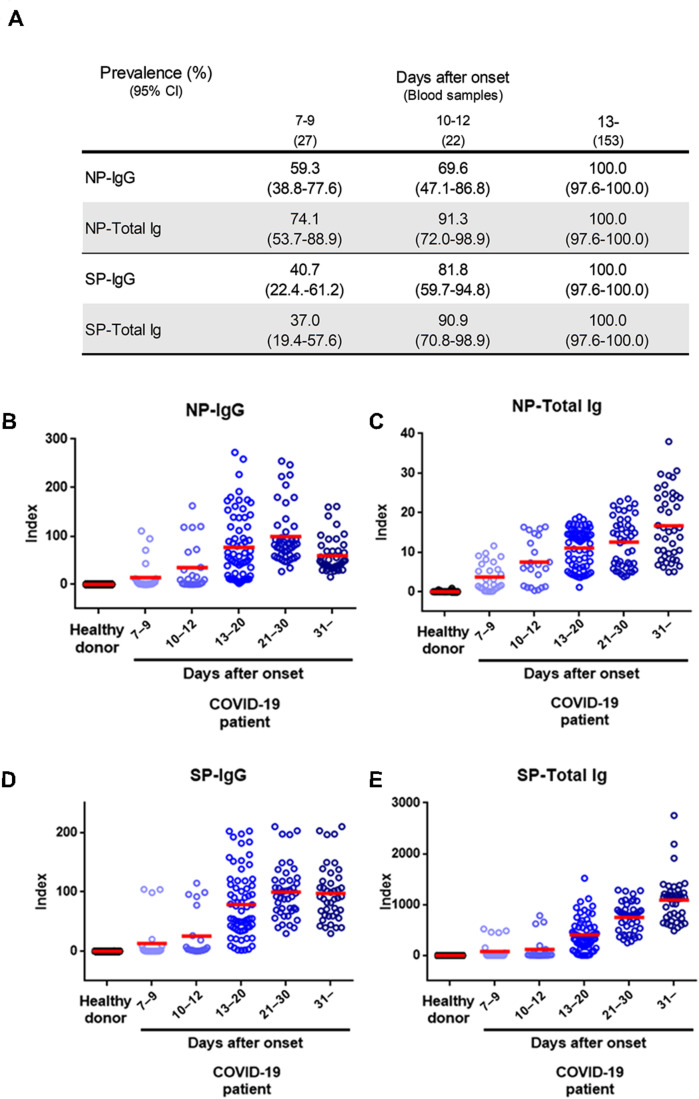
Performance characteristics of NP-IgG, NP-Total Ig, SP-IgG, and SP-Total Ig tests dedicated AIA-CL1200. **(A)** Summary of sensitivity of each antibody test at the indicated number of days after symptom onset in 202 serum samples from 44 COVID-19 patients. **(B)** Dot plots of index values of NP-IgG, **(C)** NP-Total Ig, **(D)** SP-IgG, and **(E)** SP-Total Ig at the indicated number of days after symptom onset. The index value was defined in the initial performance evaluation in Figure 2. NP, nucleocapsid protein; SP, spike protein; AIA-CL, automated chemiluminescent enzyme immunoassay system.

Next, we evaluated a total of 202 samples collected after 7 days from symptoms onset from 44 symptomatic patients with COVID-19 diagnosed by RT-PCR, using the NP-IgG, NP-Total Ig, SP-IgG, SP-Total Ig reagents. The median age of COVID-19 patients was 61.3 years in YCUH and 64.5 years in KCRC. 2 patients were mild cases, 17 were moderate cases, 16 were severe cases, 7 were critical cases, and 2 were categorized as “others” on admission (Table 1). We compared antibody titers in the following cohorts: 7–9, 10–12, 13–20, 21–30, and 31 days (up to 96 days) after onset of the disease. Clinical sensitivity of NP-IgG was 59.3% at 7–9 days, that of NP-Total Ig was 74.1%, that of SP-IgG was 40.7% and that of SP-Total Ig was 37.0%, which indicated that NP-Total Ig showed higher sensitivity than the other antibodies in early phase of COVID-19. (Figure 3A). All the four antibody titers increased around 13 days after symptoms onset, respectively (Figures 3B–E).

**TABLE 1:** Characteristics of healthy donors and COVID-19 patients.

		COVID-19 patients	
	Healthy donors	YCUH	KCRC
	n (Blood samples)	n (Blood samples)	n (Blood samples)
Gender			
Male	323 (323)	16 (161)	19 (19)
Female	677 (677)	2 (15)	7 (7)
Mean age (SD)	33.1 (7.6)	61.3 (16.5)	64.5 (17.2)
Severity			
Asymptomatic		0	0
Mild		1 (1)	1 (1)
Moderate		3 (20)	14 (14)
Severe		8 (65)	8 (8)
Critical		6 (90)	1 (1)
Others		0	2 (2)

**YCUH, Yokohama City University Hospital; KCRC, Kanagawa Cardiovascular and Respiratory Center.**

**FIGURE 4:**
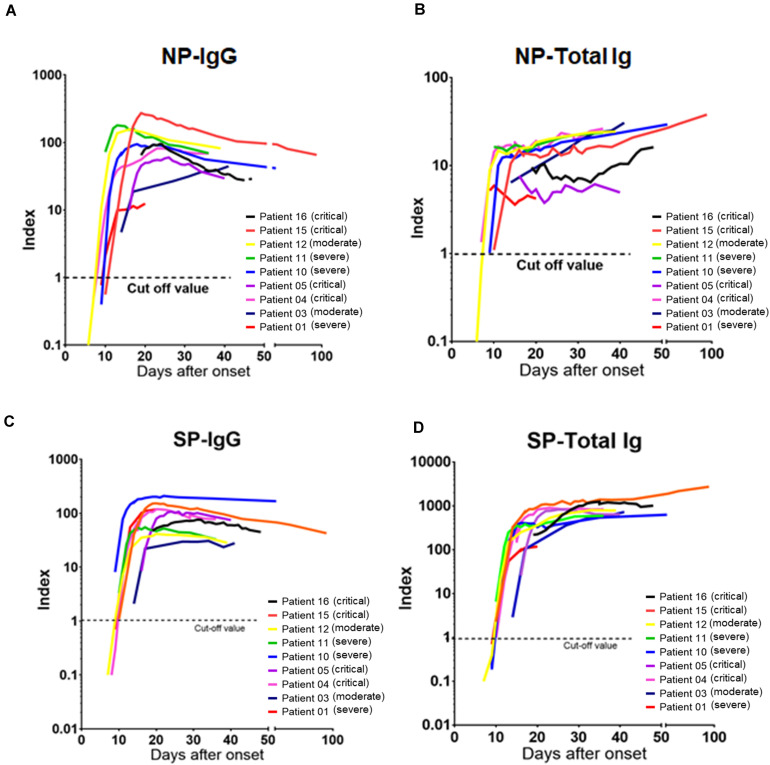
Chronological analysis of antibody titers for representative COVID-19 cases. **(A)** The time course of NP-IgG, **(B)** NP-Total Ig, **(C)** SP-IgG, and **(D)** SP-Total Ig index values from symptom onset is shown for nine individuals who received blood tests at least 5 times during administration. Each individual is represented by a different color. A 1.0 index cutoff for positivity is depicted by a horizontal dashed line. The severity is based on NIH classification. NIH, National Institutes of Health; NP, nucleocapsid protein; SP, spike protein.

Furthermore, chronological observation of antibody titers of serial samples of nine representative patients who received blood tests at least 5 times during admission are shown in Figure 4. NP-IgG titer was slightly decreased at more than 30 days after symptoms onset (Figure 4A), but NP-Total Ig, SP-IgG, and SP-Total Ig titers were increased continuously for up to 21–30 days after symptoms onset and tended to be sustained (Figures 4B–D). There was no obvious difference of the transition of each antibody titer among moderate, severe and critical cases (Figures 4A–D).

### Sensitivity and Specificity of NP-IgG, NP-Total Ig, SP-IgG, and NP-Total Ig Assays

**FIGURE 5:**
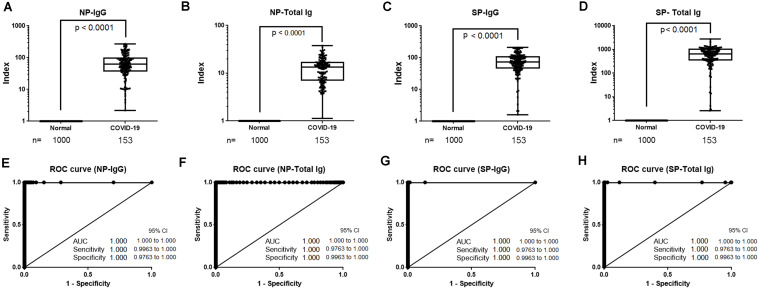
Sensitivity and specificity of NP-IgG, NP-Total Ig, SP-IgG and SP-Total Ig reagents dedicated AIA-CL1200. **(A)** Box plots for NP-IgG, **(B)** NP-Total Ig, **(C)** SP-IgG, and **(D)** SP-Total IG tests. A total of 153 samples ( > 13 days after symptoms onset) from 30 patients hospitalized in YCUH and KCRC, and 1,000 healthy donor samples were used. **(E)** ROC curves of NP-IgG, **(F)** NP-Total Ig, **(G)** SP-IgG, and **(H)** SP-Total Ig tests are shown. NP, nucleocapsid protein; SP, spike protein; ROC, receiver operating characteristic; AIA-CL, automated chemiluminescent enzyme immunoassay analyzer; YCUH, Yokohama City University Hospital; KCRC, Kanagawa Cardiovascular and Respiratory Center.

We compared antibody titers of samples from 1,000 healthy donors and 153 samples from COVID-19 patients collected 13 days after symptoms onset and analyzed using the NP-IgG, NP-Total Ig, SP-IgG, and SP-Total Ig reagents (Figures 5A–D). Subsequently, we determined the sensitivity and specificity of our antibody assays by ROC analysis. Each of the four antibodies precisely identified all the true negatives and true positives thereby achieving 100% sensitivity and specificity (Figures 5E–H). Given its excellent performance, our newly developed assay may be suitable for large-scale antibody detection.

### Correlation With Other Commercially Available Kits

We next wanted to compare the performance of our test against the commercially successful chemiluminescent Immunoassay platforms Roche-Total Ig and Abbott IgG. As these platforms utilize only the NP, we examined the correlations between the data acquired using the NP-IgG and NP-Total Ig tests from this study and the data acquired using the above mentioned commercial kits. For this purpose, we used 29 COVID-19 antibody–positive samples. Results from NP-IgG were strongly correlated with the results from Roche NP-Total Ig (Spearman’s correlation coefficient, *r* = 0.97) and Abbott NP-IgG (*r* = 0.962), as were the results from NP-Total Ig (*r* = 0.989 and 0.875, respectively) (Table 2). Because our antibody tests can measure both NP-IgG and NP-Total Ig with high reliability and additionally SP-IgG and SP-Total Ig, they have a practical scope for COVID-19 serological analysis.

**TABLE 2:** Correlations with positivity rates of other commercially available kits for COVID-19 antibody using Spearman’s correlation coefficient.

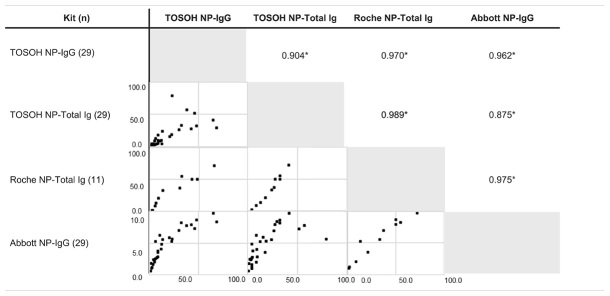

NP, nucleocapsid protein.

*: p < 0.001.

## Discussion

COVID-19 being a global public health problem, surveillance to estimate infection rates based on percentage of antibody positivity and chronological analysis of antibody titers are actively being conducted (Gudbjartsson et al., 2020). This is done either as a qualitative assessment using a rapid diagnostic kit or as a quantitative evaluation by antibody titer measurement using ELISA. In particular, the large-scale evaluation of seroprevalence and herd immunity against SARS-CoV-2 requires a high-throughput and quantitative screening as it is more rapid and accurate measurement of viral antibody levels. In this study, we established a combination of NP-IgG, NP-Total Ig, SP-IgG, and SP-Total Ig tests dedicated AIA-CL1200. The sensitivity and specificity of each of the four antibody assay individually was 100% in COVID-19 patient samples collected 13 days after symptoms onset. Moreover, the NP-IgG and NP-Total Ig assays correlated strongly with Abbott and Roche assays for COVID-19 serodiagnosis (Bryan et al., 2020; Lau et al., 2020). Considering its excellent performance, this assay could play important roles in generating authentic data during public surveillance of COVID-19.

All the four types of antibody titers began to rise 7–9 days after symptoms onset and were positive in 100% of patients after 13 days, which is consistent with previous reports (Burbelo et al., 2020; Hou et al., 2020; Long et al., 2020a). In addition, antibodies against NP had higher sensitivity than antibodies against SP in the early phase of COVID-19 infection and NP-Total Ig showed the highest sensitivity among the four antibodies. It has been reported that the positivity of NP antibodies is higher than SP antibodies in acute phase of SARS-CoV-2 infection (Borremans et al., 2020; Burbelo et al., 2020). As in common viral infections, COVID-19 infection also has polyclonal elevation of Immunoglobulin such as IgM and IgA, not only IgG. Although many antibody kits can measure only IgG or/and IgM, it has been reported that measuring IgA in addition to IgG or IgM increases the sensitivity of the assay (Ma et al., 2020). Since our assay measures total-Ig in addition to IgG, it can possibly be more precise than assays that only detect a particular Ig type.

Antibodies against spike antigen, especially RBD, have the ability to prevent the entry of the virus into the host cells (Noy-Porat et al., 2020; Tian et al., 2020). According to previous reports, while NP-IgG declined slowly over the course of approximately half a year and turned negative in some cases, SP-IgG declined slowly but sustained its antibody titer and rarely turned negative (Ripperger et al., 2020). In this study, all the four antibodies were positive in all cases after 13 days from symptoms onset, and NP-Total Ig, SP-IgG, and SP-Total Ig have been sustained during the period of observation. However, NP-IgG titers tended to decrease after 30 days from symptoms onset (Figure 4) similar to the trend noted by Long et al., who observed the decline of IgG antibodies in convalescence (Long et al., 2020b). Multiple antibody measurements, especially antibodies against SP, are likely to be useful for assessment of long-term changes of antibody titers and providing important insights into immune memory.

It should be noted that there were some limitations in the study. First, most of the cases included in this study were of high severity. It is known that COVID-19 antibody titers tend to be lower in less severe cases (Long et al., 2020b; Lynch et al., 2020). Although we did not find any obvious differences in the antibody titers between moderate, severe and critical cases in this study, it was insufficient to evaluate the difference in variation of antibody titers with the spectrum of severity of COVID-19 because there were only few samples obtained from mild cases and none from asymptomatic infections. This was because of the inherent difficulty in obtaining these samples as mild infections are usually home quarantined without serological testing and the asymptomatic infections go unidentified. Therefore, there is a possibility that the cutoff value of the assay set in this study may not be appropriate for detecting the lower antibody titers occurring in less severe cases, such as mild or asymptomatic infections. Second, the newly developed reagents can only measure IgG and total Ig, but not other specific immunoglobulin subclasses, such as IgM or IgA. This could result in the sera with large amounts of IgM and/or IgA titers giving off higher signals, resulting in non-linear and/or non-quantitative signals due to their multimerization properties. Also, this study showed that NP-Total Ig was sustained but NP-IgG decreased after 30 days since symptoms onset. The reason for this reduction in IgG titers is unclear and the other immunoglobulin which elevated to sustain the total Ig levels also remains to be identified with further investigations.

## Conclusion

In conclusion, our current assay system enables to measure 4 different types of antibodies (IgG and total Ig for SP and NP antigens) simultaneously for a single sample within 15 min with high accuracy and specificity. Furthermore, with the ability of running 120 tests/h with the AIA-CL1200 device, the assay can provide high throughput results. Therefore, our assay system can offer an advantage in performing large-scale sero-surveillance studies allowing for the detailed understanding of chronological changes and individual immune status.

## Data Availability Statement

The datasets presented in this article are not readily available because they contain potentially sensitive patient information. This policy follows the restrictions imposed by Yokohama City University Hospital and Kanagawa Cardiovascular and Respiratory Center institutional review board that approved the study. Electronic health record data cannot be shared publicly because they consist of personal information from which it is difficult to guarantee de-identification. As a result, there is a possibility of deductive disclosure of participants and therefore full data access through a public repository is not permitted by the institutions that provided us the data. The data and associated documentation from each collaborating institution can only be made available under a new data sharing agreement with which includes: (1) commitment to using the data only for research purposes and not to identify any individual participant; (2) a commitment to securing the data using appropriate measures, and (3) a commitment to destroy or return the data after analyses are complete. Requests to access the datasets should be directed to AR, aryo@yokohama-cu.ac.jp.

## Ethics Statement

The studies involving human participants were reviewed and approved by the Yokohama City University Hospital and Kanagawa Cardiovascular and Respiratory Center institutional review board. Written informed consent was not provided because All patients were recruited using the opt-out approaches.

## Author Contributions

SK and NO analyzed the data, verified the analytical methods, and wrote the manuscript. SJ and KM analyzed the data and wrote the manuscript. YY, TM, AG, TK, and TY analyzed the data. KM, EH, EY, and TO contributed reagents. AR directed the research, analyzed the data, and wrote the manuscript. All authors contributed to the article and approved the submitted version.

## Conflict of Interest

At the time of conducting the sudy, NO is a current employee of Tosoh Corporation and YY is a current employee of Kanto Chemical Co., Inc. The remaining authors declare that the research was conducted in the absence of any commercial or financial relationships that could be construed as a potential conflict of interest.

## Supplementary Material

Supplementary Figure 1 Intensity counts of the chemiluminescent signals of SP-Total Ig in the AIA-CL according to the concentration of immobilized SP antigen on micro-magnetic beads. To evaluate the optimization of the SP antigen amounts for measuring SP-Total Ig, the immobilized SP antigens on micro-magnetic beads were assigned to 0.005, 0.01, and 0.30 mg/dl, and intensity counts of the chemiluminescent signals of SP-Total Ig were measured by AIA-CL. Welche’s *t*-test was used to compare the mean values of the samples of healthy donors’ serum and those of COVID-19 patients’ serum. Green bars indicate the mean value of each group. The intensity counts of chemiluminescent of two negative samples were higher than the other negative samples at a concentration of 0.030. SP, spike protein; AIA-CL, automated chemiluminescent enzyme immunoassay analyzer; Pos, the sample of COVID-19 patients’ serum; Neg, the sample of healthy donors’ serum.

Supplementary Figure 2 Evaluation of linearity of NP-IgG, NP-Total Ig, SP-IgG and SP-Total Ig in AIA-CL1200. To evaluate the linearity of the assay, a two-fold serial dilution of COVID-19 patient’s serum was used and **(A**,**D)** NP-IgG, **(B,E)** NP-Total Ig, **(C,F)** SP-IgG, and **(D,G)** SP-Total Ig were measured in the AIA-CL. The ratio of the intensity counts of chemiluminescence signals to serum dilution concentration (gradient value) was calculated for each sample. NP, nucleocapsid protein; SP, spike protein; AIA-CL, automated chemiluminescent enzyme immunoassay analyzer.
