# Supplementary figures and images for: Development of an Automated Chemiluminescence Assay System for Quantitative Measurement of Multiple Anti-SARS-CoV-2 Antibodies
Source: Front Microbiol. 2021 Jan 15;11:628281. doi: 10.3389/fmicb.2020.628281 (PMC7843449; doi:10.3389/fmicb.2020.628281)

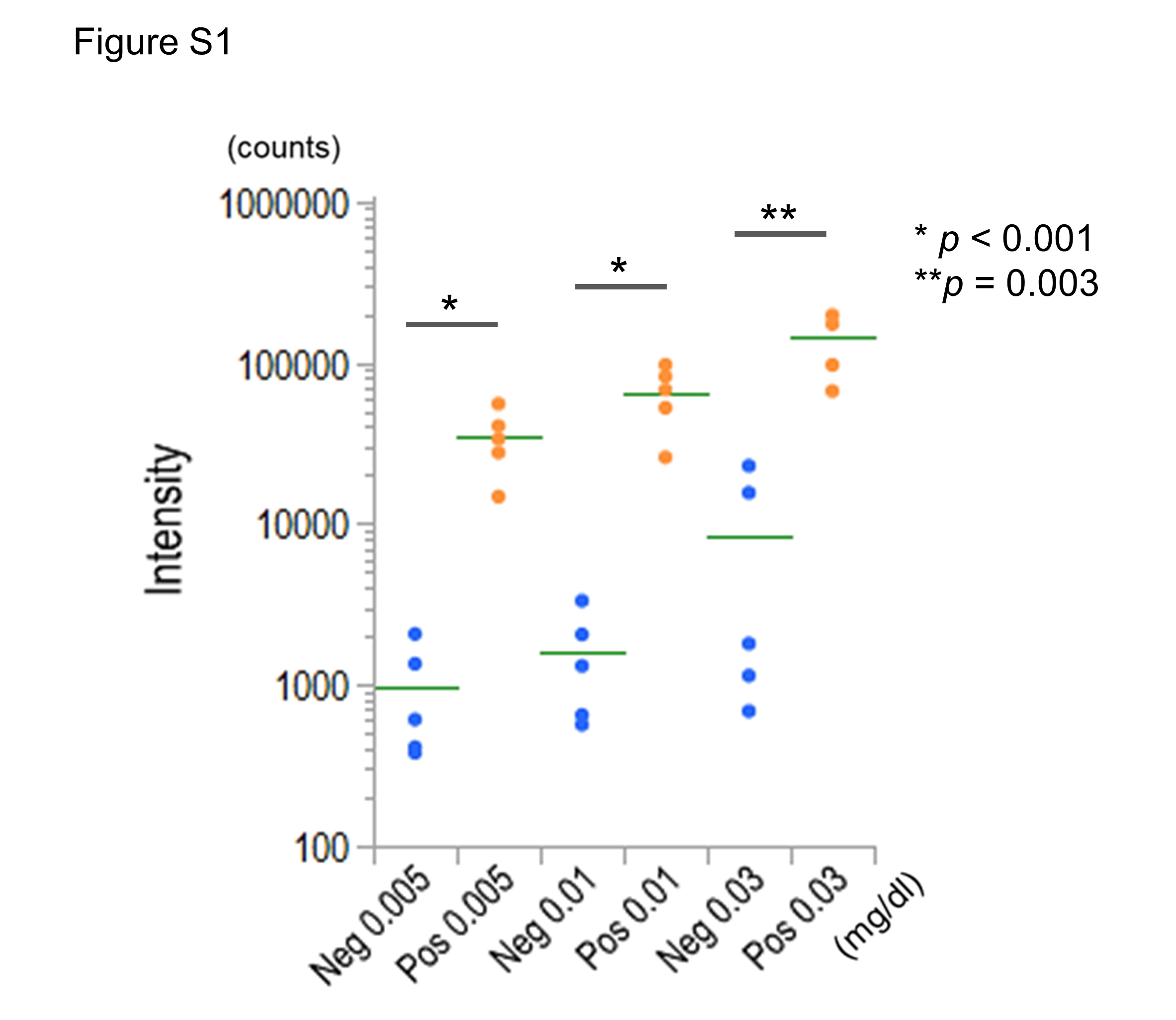

Supplement: Supplementary Figure 1 — Intensity counts of the chemiluminescent signals of SP-Total Ig in the AIA-CL according to the concentration of immobilized SP antigen on micro-magnetic beads. To evaluate the optimization of the SP antigen amounts for measuring SP-Total Ig, the immobilized SP antigens on micro-magnetic beads were assigned to 0.005, 0.01, and 0.30 mg/dl, and intensity counts of the chemiluminescent signals of SP-Total Ig were measured by AIA-CL. Welche’s t-test was used to compare the mean values of the samples of healthy donors’ serum and those of COVID-19 patients’ serum. Green bars indicate the mean value of each group. The intensity counts of chemiluminescent of two negative samples were higher than the other negative samples at a concentration of 0.030. SP, spike protein; AIA-CL, automated chemiluminescent enzyme immunoassay analyzer; Pos, the sample of COVID-19 patients’ serum; Neg, the sample of healthy donors’ serum. [file Image_1.TIF]

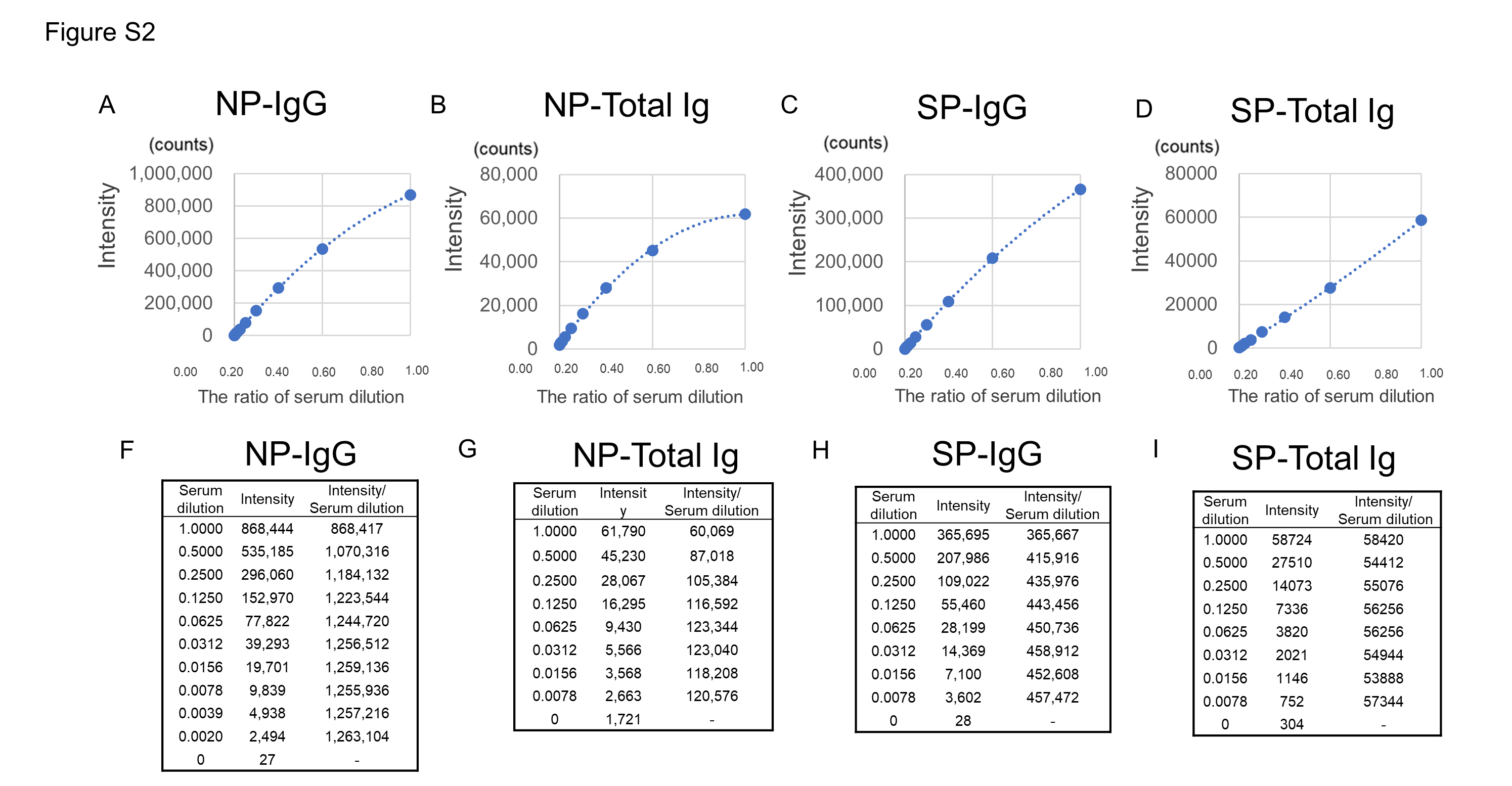

Supplement: Supplementary Figure 2 — Evaluation of linearity of NP-IgG, NP-Total Ig, SP-IgG and SP-Total Ig in AIA-CL1200. To evaluate the linearity of the assay, a two-fold serial dilution of COVID-19 patient’s serum was used and (A,D) NP-IgG, (B,E) NP-Total Ig, (C,F) SP-IgG, and (D,G) SP-Total Ig were measured in the AIA-CL. The ratio of the intensity counts of chemiluminescence signals to serum dilution concentration (gradient value) was calculated for each sample. NP, nucleocapsid protein; SP, spike protein; AIA-CL, automated chemiluminescent enzyme immunoassay analyzer. [file Image_2.TIF]
